# Matters arising: bridging predictive algorithms and clinical practice in DBS contact selection

**DOI:** 10.1038/s41531-026-01496-4

**Published:** 2026-08-27

**Authors:** Annabel van der Weide, Yarit Wiggerts, Deborah Hubers, Bart J. Keulen, Martijn G. J. de Neeling, Mariëlle J. Stam, Bernadette C. M. van Wijk, Maarten Bot, Rick Schuurman, Rob M. A. de Bie, Martijn Beudel

**Affiliations:** 1 Department of Neurology, Amsterdam University Medical Center, Amsterdam Neuroscience, University of Amsterdam, Amsterdam, Netherlands; 2 Department of Neurosurgery, Amsterdam University Medical Center, Amsterdam Neuroscience, University of Amsterdam, Amsterdam, Netherlands; 3 Department of Human Movement Sciences, Faculty of Behavioural and Movement Sciences, Vrije Universiteit Amsterdam, Amsterdam, Netherlands

**Keywords:** Biomarkers, Computational biology and bioinformatics, Engineering, Neuroscience

**arising from** Marjolein Muller et al. *npj Parkinson’s Disease* https://doi.org/10.1038/s41531-025-01092-y (2025)

Optimal contact selection is a critical determinant of deep brain stimulation (DBS) outcome in Parkinson’s disease, yet it remains one of the most challenging steps in clinical practice. When the optimal stimulation contact is identified directly, precise electrical parameters can be applied without exhaustive testing at every individual electrode contact, saving clinical time and reducing patient burden^1^. Conversely, suboptimal contact selection carries consequences that extend well beyond inefficiency: when the therapeutic target is missed, side effects become more likely. In search of adequate effect, clinicians are then compelled to explore a wide range of contacts and parameter combinations, exposing patients to repeated adjustments, conflicting sensations, and the confusion and overload that comes with prolonged and inconclusive programming trajectories. Neural biomarkers, specifically local field potentials (LFPs) recorded from chronically implanted DBS devices, offer a promising route toward network-guided, patient-specific programming. By revealing the spatial distribution of pathological beta band ( ± 13–35 Hz) activity, LFPs could enable direct identification of the optimal contact and, ultimately, support adaptive DBS systems that continuously adjust stimulation to the patient’s momentary neural state. Realizing this potential, however, requires that LFP-based contact selection algorithms are not only accurate but robust and reproducible across clinicians and centers, a standard that has yet to be rigorously established.

In their recent study, Muller et al. analyzed LFP recordings to predict the optimal contact for stimulation, which could potentially reduce the burden of programming for both patient and clinician^2^. The authors retrospectively analyzed bipolar LFP recordings from multiple centers and developed two predictive algorithms: a decision tree method for in-clinic use and a pattern-based method for offline validation. Both methods achieved promising accuracies (up to approximately 87% across centers) when considering the top two predicted contact levels, outperforming an existing algorithm^3^ that is used for the same application. While these results contribute to the integration of neurophysiological biomarkers into DBS programming workflows, translating predictive accuracy into robust, standalone clinical decision tools remains difficult. Specifically, Muller et al.’s approach focuses on ranking a set of the best candidate stimulation contacts based on features of beta-band power derived from bipolar LFP recordings. To better understand the clinical relevance of the decision tree approach, we evaluated its performance at two levels: first, its ability to identify the single optimal stimulation contact, and second, whether it provides additional value over simple anatomical heuristics that are commonly used in clinical practice.

We performed a follow-up analysis in our center where we implemented the published decision tree algorithm on an independent dataset (51 hemispheres). The algorithm generated two candidate contacts per hemisphere. From these two options, three independent raters were asked to select the single optimal contact based on visual inspection and expert judgment. Representative examples are shown in Fig. 1. Raters were selected based on prior experience with DBS programming and analysis of LFP recordings. For each hemisphere, raters were provided with the bipolar BrainSense^TM^ Survey recordings and reviewed the distribution of beta-band activity across available bipolar contact pairs. No additional imaging or clinical outcome data were provided. Agreement for the top 1 contact was limited. Pairwise Cohen’s κ ranged from −0.02 to 0.46, corresponding to no to moderate agreement depending on the rater pair, with an overall Fleiss’ κ of 0.24 across all three raters. These findings suggest that, despite effective narrowing of potentially optimal contacts, considerable ambiguity remains when choosing the final contact for chronic stimulation based on LFP recordings. All raters had prior experience LFP interpretation and worked within the same center, where regular clinical and research interactions resulted in a shared approach to electrophysiological signal interpretation. Although no formal calibration session was performed before the study, the variability observed is therefore unlikely to be explained solely by differences in expertise or familiarity with LFP analysis. To understand why raters disagreed, we reviewed the cases where they reached different conclusions. The root cause lies in a fundamental property of how DBS devices record LFP signals. Rather than measuring activity at a single contact point, recordings are always bipolar: the device captures the difference in electrical activity between two contacts simultaneously. A high beta peak in, for example, the 1–3 channel indicates a strong signal difference between those two contacts, but does not reveal which of the two is actually sitting closest to the beta source. It could be contact 1, contact 3, or even contact 2 sitting in between them. When faced with this ambiguity, some raters assumed the dominant activity came from one of the two recording contacts, while others assumed it came from the contact in between. Both interpretations are physiologically plausible, yet they lead to different contact recommendations. Crucially, the decision tree provides no explicit rule to resolve this choice, meaning that a step requiring clinical judgment remains embedded within what appears on paper to be an objective algorithm. It is precisely at this unspecified interpretative step that rater disagreement arose.

**Fig. 1:**
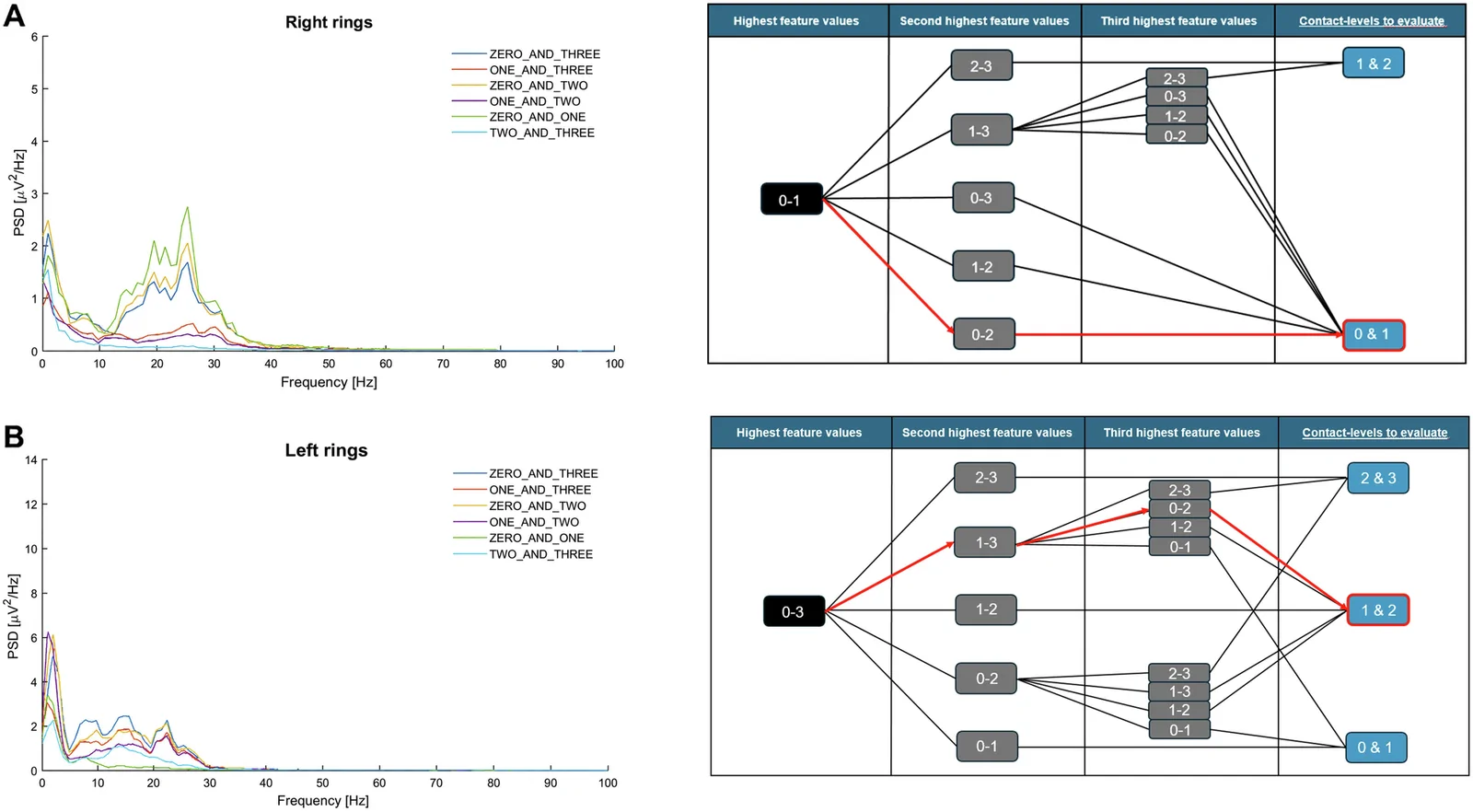
Representative examples of DBS contact selection using the decision tree proposed by Muller et al. The left panels show the power spectral density (PSD) of all bipolar contact pairs. The right panels show the decision tree, which reduces the available contacts to two candidate contacts for stimulation. Red pathways indicate the branch followed based on the beta-power features. The original decision tree was adapted from Muller et al. and highlighted pathways were added by the authors. **A** **Agreement between raters.** The decision tree identified contacts 0 and 1 as candidate contacts. All raters selected contact 0 as the optimal stimulation contact because beta activity was consistently stronger in bipolar pairs containing contact 0, while the other pairs containing contact 1 (i.e., 1–2 and 1–3) showed minimal or absent beta peaks. **B** **Disagreement between raters.** The decision tree identified contacts 1 and 2 as candidate contacts. One rater selected contact 2 as the optimal stimulation contact, arguing that the largest activity was found in the contact pairs containing contact 3, suggesting maximal activity is located more dorsally, therefore choosing contact 2 over contact 1. Two raters selected contact 1, arguing that bipolar pair contact 1^1–3^ exhibited greater beta power than the corresponding pair containing contact 2^2,3^.

We further evaluated whether the decision tree approach provided additional predictive value in narrowing the set of candidate stimulation contacts, by comparing it to a simple anatomical heuristic based on typical lead positioning: selecting the two middle contacts (contacts 1 and 2), which are commonly located within the motor subthalamic nucleus (STN) representing the primary therapeutic target^4^. In our center, 7 T MRI- tractography guided targeting combined with intraoperative CT verification ensures that the middle contacts are by design positioned within this therapeutic target^5^, and imaging-based confirmation of lead position is considered an essential first step before any contact selection procedure is undertaken. We first compared both approaches in their ability to identify the imaging-defined optimal contacts. In our dataset, selecting either of these middle contacts achieved a predictive accuracy of 0.80, which was similar to the accuracy achieved by the decision tree (0.82). Similarly, when predicting the clinically selected stimulation contact, no significant difference was observed between the decision tree (accuracy 0.94) and the predefined middle contacts (accuracy 0.98; McNemar test *p* = 0.21).

Together, our ancillary findings highlight two key observations. First, despite narrowing the set of candidate contacts, substantial ambiguity remains when selecting a single optimal contact for chronic stimulation. Second, the decision tree approach does not substantially outperform a simple anatomical heuristic based on typical lead positioning. Several factors may contribute to these findings. An inherent limitation of neurophysiology-based contact selection strategies is their dependence on the presence of a measurable signal. The proposed decision tree relies on identifying beta-band peaks in bipolar LFP recordings. However, in clinical practice, such peaks may be absent or poorly distinguishable in a subset of patients, limiting the applicability of the method. Although beta oscillations are considered a marker of Parkinsonian symptom severity, their expression differs depending on symptom expression and recording conditions^6^. This means that beta activity does not always provide a clear or consistent signal for selecting the optimal stimulation contact. As a result, algorithms that rely on fixed spectral features may perform inconsistently across patients. In our dataset, a subset of hemispheres showed no clear dominant beta peak. Although the method by Muller et al. partially addresses this by ranking candidate contacts, ambiguity remains when selecting a single contact for chronic stimulation. Furthermore, LFP-based physiomarkers are primarily associated with motor symptoms or dyskinesia^7^ and less with stimulation-induced side effects such as neuropsychiatric sequelae, dysarthria or capsular activation. In clinical practice, contact selection requires balancing therapeutic benefit against adverse effects, which cannot be fully inferred from electrophysiological features alone. Future work should therefore focus not only on predicting therapeutic efficacy but also on quantifying the anatomical and electrophysiological determinants of stimulation-induced side effects.

Next to this, the 7T MRI level of anatomical precision we used for our analyses is not standard practice worldwide, and in centers where targeting accuracy is less certain, the added value of LFP-based contact selection may be considerably greater. The present comparison, therefore, does not argue against LFP guidance in general but rather suggests that its incremental value is closely tied to the quality of the underlying lead placement. As a consequence of this targeting precision, contacts 1 and 2 represent the anatomically optimal stimulation location in the large majority of our implantations. Critically, beta-band LFP activity is strongest precisely where the electrode is most accurately placed within the motor STN, meaning that in a well-targeted implantation, LFP-based ranking and anatomy-based selection will converge on the same contacts by definition. Both approaches, therefore, arrive at the same recommendation through different routes, but for the same underlying reason: accurate targeting has already determined the answer before either method is applied. Ultimately, DBS outcomes arise from a complex interaction between electrode location, network anatomy, stimulation parameters, and individual patient physiology. Therefore, physiomarkers derived from LFP recordings should be interpreted together with imaging and clinical outcome measures, rather than used in isolation.

The growing availability of sensing-enabled DBS systems provides new opportunities to integrate electrophysiological information into programming strategies. Our ancillary analyses support the notion that LFP-based algorithms such as the decision tree algorithm by Muller et al. can potentially reduce the search space during DBS programming by identifying a pair of candidate contacts. This is consistent with the intended purpose of the algorithm, namely, to reduce the search space rather than to directly determine a single optimal contact. In this sense, the top-2 prediction may represent a clinically appropriate endpoint of the algorithm, as also suggested by Muller et al. Our findings do not challenge this approach; rather, they highlight the next unresolved step in clinical decision-making. Translating such a reduced set of candidate contacts into the reliable selection of a single optimal stimulation contact. Our findings suggest that variability in interpreting LFP-derived beta peak power features may limit the robustness of algorithm-based contact selection. Importantly, in datasets where electrode placement is accurate, simple anatomical heuristics may perform similarly to more complex LFP-based approaches.

An additional consideration is the increasing use of directional DBS contacts. Both the algorithm proposed by Muller et al. and the anatomical heuristic evaluated in the present study operate at the contact-level rather than the segment-level. In directional leads, however, multiple segmented contacts increase the complexity of contact selection. Consequently, the present findings should be interpreted within the context of contact-level selection. Future studies should evaluate whether LFP-based prediction methods can facilitate segment-level programming in directional DBS electrodes. Another important consideration is that the present findings relate to bipolar LFP recordings, which remain the most widely available sensing modality and form the basis of the algorithm evaluated in this study. Emerging sensing-enabled DBS systems now also permit pseudo-monopolar LFP recordings referenced to the electrode of the contralateral hemisphere. Although clinical evidence regarding their utility remains limited, such recordings may provide more contact-specific estimates of neural activity and could potentially reduce the ambiguity inherent to bipolar recordings. Future studies should investigate whether this approach improves the robustness and reproducibility of LFP-guided contact selection. Another important direction for future research is to better define which patients are most likely to benefit from electrophysiology-guided contact selection and to further explore LFP features besides beta peak power for improving LFP-based contact selection^8^. In line with current evidence^2,3^, our study suggests that algorithms based on LFP features are best viewed as decision-support systems rather than replacements for clinical judgment. Further progress of LFP-based algorithms will likely involve prospective validation of predictive models, iterative refinement of algorithms based on clinical outcomes, and the development of multimodal frameworks that combine electrophysiology with anatomical and clinical information.

In this sense, predictive LFP-based algorithms for contact selection should be viewed as part of an evolution in DBS programming: a transition from exhaustive testing toward a more data-informed approach in which electrophysiological and anatomical information are integrated to enable patient-specific, network-guided stimulation.

## Data Availability

All data supporting the findings of this study are available upon reasonable request.
